# A natural heparinoid from mollusc *Meretrix lusoria*: Purification, structural characterization, and antithrombotic evaluation

**DOI:** 10.1016/j.crfs.2022.10.010

**Published:** 2022-10-07

**Authors:** Jing Chen, Zhenxing Du, Bingbing Song, Rui Li, Xuejing Jia, Jianping Chen, Xiaofei Liu, Saiyi Zhong

**Affiliations:** a College of Food Science and Technology, Guangdong Ocean University, Guangdong Provincial Key Laboratory of Aquatic Product Processing and Safety, Guangdong Province Engineering Laboratory for Marine Biological Products, Guangdong Provincial Engineering Technology Research Center of Seafood, Zhanjiang, 524088, China; b Shenzhen Research Institute, Guangdong Ocean University, Shenzhen, 518108, China; c Collaborative Innovation Center of Seafood Deep Processing, Dalian Polytechnic University, Dalian, 116034, China

**Keywords:** Heparinoid, Shellfish, Antithrombotic activity, Hemorrhagic effect

## Abstract

Heparinoid, a sulfate polysaccharide derived from marine organisms was attracted largely attention due to its versatile activities. A naturally occurring heparinoid (M2) that was extracted from the mollusk *Meretrix lusoria* and used in this investigation shown strong antithrombotic action. UV–Vis, FT-IR, SAX-HPLC, and NMR were used to explore the structural characteristics of M2, results indicated that M2 similar with heparin, its average molecular weight was 22.58 kDa. Which was primarily made up of→4)-α-IdoA2S-(1→4)-α-GlcNS6S-(1→ (31.19%), →4)-β-GlcA-(1→4)-α-GlcNAc (1→ (23.21%), →4)-β-GlcA-(1→4)-α-GlcNS (1→ (13.87%), →4)-α-IdoA2S-(1→4)-α-GlcNS (1→ (8.95%), →4)-β-GlcA-(1→4)-α-GlcNAc6S (1→ (7.39%) and →4)-β-GlcA-(1→4)-α-GlcNS6S (1→ (7.63%). The antithrombotic activity of M2 was evaluated using measurements of the anticoagulant effect *in vitro* and the fibrinolytic capability *in vitro* and *in vivo*, and M2 has 122.4 U/mg of anticoagulant activity and 1.41 U/mg of fibrinolytic activity, respectively. Additionally, a mouse tail-cutting model was used to assess the bleeding effect in real time, it found that M2 had a reduced hemorrhagic risk than heparin. Consequently, M2 could be exploited to develop functional foods or medications with antithrombotic properties.

## Highlights

• A natural heparinoid (M2) was discovered in the seafood Meretrix lusoria.
• M2 has slightly lower anticoagulant activity than mammalian heparin but a weaker bleeding effect.
• M2 was found to have an active effect on the fibrinolysis pathway in vitro and in vivo.
• M2 considered replacing mammalian heparin with functional foods or drugs with anti-thrombotic effects.

## Graphical abstract

**Figure:**
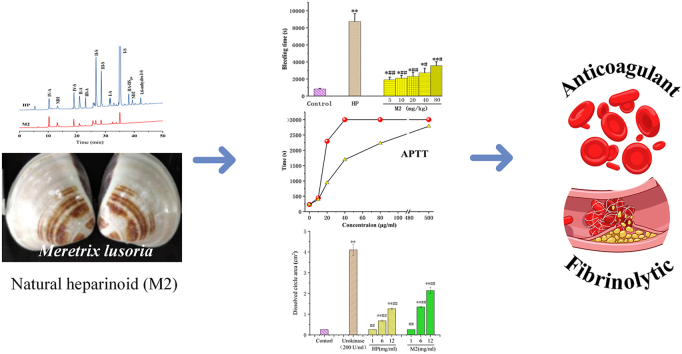

## 1 Introduction

Nowadays, thrombotic diseases have been severely affecting human health (Micco, 2022) and even become one causes of morbidity and mortality high around the world (Vazhappilly et al., 2019). Additionally, the coronavirus disease 2019 (COVID-19) pandemic exposed patients vulnerable to thrombotic and thromboembolic events (Gorog et al., 2022). Thrombosis is attributed to the formation of blood clot within the blood vessel, such as hypercoagulability. There is a dynamically balanced between coagulation system and fibrinolytic system in human body, which makes the blood flow smoothly. Commonly, antithrombotic drugs, such as anticoagulants and fibrinolytic drugs, there is accurately effective for preventing thrombosis and dissolving thrombus. It has been thought that anticoagulant could play a critical role before thrombosis. For initial formation of thrombus, the fibrinolytic system of body would be immediately activated and release fibrin simultaneously, which dissolved thrombus. Hence, the research on prevent thrombotic disease has become one of the studies focuses.

Heparin has been widely used as an anticoagulant drug in treating cardiovascular diseases for over 90 years (Shan and Ningchuan Feng, 2019). Heparin, a highly sulfated glycosaminoglycan possessed strong surface charge and alternating D-glucosamine and hexuronic acid residues (Qiu et al., 2021). In addition, previous studies showed that heparin also showed antithrombotic, antioxidant, antiviral, anti-tumor and anti-inflammatory activities (Hippensteel et al., 2020; Mulloy, 2019; Qiu et al., 2021). However, the clinical application of heparin for treatment is limited by several drawbacks, such as limited as raw material source and severe anticoagulant activity that could induce hemorrhaging (Cheng et al., 2017). Commonly, heparin was primarily derived from pig intestine and bovine lung, at the same time, the bovine and porcine heparin were affected by African swine fever and the possible bovine prion disease to humans (Flengsrud et al., 2010). Therefore, it is necessary to exploration the alternative heparin with prevention and treatment of thrombosis. Marine bioactive compounds have great potential in the treatment of thrombosis, which attracts much attention. Many studies have reported that marine heparin exhibit pretty good coagulation such as derive from *Tridacna maxima* and *Perna viridis* (Arumugam and Giji, 2014), crabs (Andrade et al., 2013), shrimp head (Chen, 2021). Beyond that, ascidian heparin showed stronger antithrombotic activity than mammalian heparin in arterial animal models, its bleeding effect is lower (Santos et al., 2007). In contrast to mammalian heparin, there has been plentiful research showing that marine heparin is obvious to exhibit a less hemorrhagic effect and decreased side effects in patients (Brito et al., 2014; Colliec-Jouault et al., 2012). Currently, clam heparin has received considerable interests because of its convenient isolation, potent biological activities and available raw materials (Du et al., 2018; Du et al., 2019a, 2019b).

**Fig. 1:**
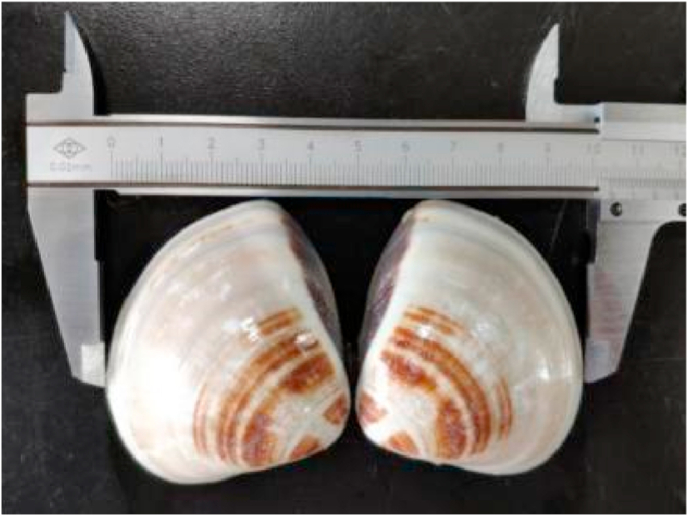
*Meretrix lusoria* from Zhanjiang Dongfeng Market, China.

*M. lusoria* (Fig. 1), a rich source of nutritious seafood with low price and readily available in China, now it had attracted wide attention due to its various bioactivities by reducing blood lipid, anti-mutation, anti-aging, and immunoregulation (Liu, Z Y et al., 2018). According to Wang, glycosaminoglycans constitute a little over 4% of total glycan components in clam tissues and consists primarily of amino sugars and uronic acid (Wang, L et al., 2018). An earlier study reported that glycosaminoglycan prepared from *Meretrix meretrix* might be an alternative source of heparin (Saravanan et al., 2010). Although studies on heparin from shellfish have been conducted, the heparin from *Meretrix Lusoria* and its biological activity have yet to be investigated. The aims of this work is to isolate a natural heparinoid from *M. lusoria*, and evaluate its hemorrhagic effect, anticoagulant activity and fibrinolytic activity. This study provided a timely and necessary basis for further development of marine heparin and antithrombotic polysaccharides. It provides a theoretical basis for utilizing and developing marine heparinoids.

## 2 Materials and methods

### 2.1 Reagents

*M. lusoria* was obtained from Zhan Jiang, China. Plasminogen, dermatan sulfate (DS), chondroitin sulfate (CS), and Mammalian heparin (HP) were purchased from the National Institutes for Food and Drug Control (Beijing, China). Rabbit plasma, bovine plasma fibrinogen, urokinase (50000 U/g), thrombin (300 U/mg), alkaline protease 2709 and papain were gifts from Shanghai yuanye Bio-Technology Co. Ltd (Shanghai, China). Thrombin time (TT), prothrombin time (PT) and activated partial thromboplastin time (APTT) test kits were obtained from Sun Bio-Technology Co. Ltd. (Shanghai, China). Urokinase-type plasminogen activator (u-PA), tissue-type plasminogen activator (t-PA), and plasminogen activator inhibitor (PAI-1) were obtained from Shanghai Lengton Bioscience Co., Ltd (Shanghai, China). Standard monosaccharide references were prepared from Sigma-Aldrich (Germany). Heparin standards (Mw: 3500, 5000, 8000, 15000, and 30000 Da) were obtained from Adhoc Bio-Technology Co., Ltd (Beijing, China). Heparinlyase I, II, III were purchased from Asnail Biotechnology Co. Ltd. (Beijing, China). Unless specified otherwise, all chemicals were of analytical grade or HPLC grade.

### 2.2 Animals

Sprague-Dawley (SD) female rats, weighing 220–250 g, were acclimated to the laboratory for at least 7 days prior to surgery or experimentation. All experiments were carried out according to the NIH. (Bioethical Committee approval no. SYXK2014-0053).

### 2.3 Isolation heparinoid of M. lusoria

The clam *M. lusoria* was obtained from Zhanjiang Dongfeng Market, China. Dried tissue was obtained after homogenizing the soft tissue and freeze-drying. 100 g of this dry tissue were mixed with 10 times volume distilled water in high-speed tissue masher, and pH adjusted to 8.0. Then, NaCl (6 mg/mL), Papain (5.4 mg/mL) and 2709 alkaline protease (5.4 mg/mL) were put in and hydrolyzed for 20 h (50 °C). Next, the hydrolysate was boiled for 10 min, centrifuged (8000 r/min, 30 min) after cooling, and the supernatant was obtained. The supernatant was transferred to a column (26 cm × 100 cm) filled with AMBERLITE FPA98 Cl macro porous ion exchange resin. Dynamic adsorption was carried out by constant flow pump at 45 °C, and the adsorption rate was 10 mL/min. After adsorption, gradient elution was performed with 0, 1.0, 1.5, and 3.5 mol/L NaCl solution in sequence. Determination of glycosaminoglycan content in each fraction by Alcian blue method. Fractions eluted with 1.0 mol/L, 1.5 mol/L, and 3.5 mol/L NaCl were collected and precipitated by 0.4 vol of ethanol overnight. The precipitate was collected by centrifugation, dissolved in distilled water, dialyzed for 48 h at 4 °C, and lyophilized. The elution components of 1.0 mol/L, 1.5 mol/L, and 3.5 mol/L NaCl were named M1, M2, and M3, respectively.

### 2.4 Physicochemical properties analysis of fractions

Heparin anticoagulant potency was evaluated by comparing with the concentration necessary to prevent the clotting of rabbit plasma using CHP (Chinese Pharmacopoeia) method, and heparin standard (197 U/mg) was used as control; Heparin content was determined by methylene blue spectrophotometry with heparin as standard; Protein content was measured by Folin-phenol method with bovine serum protein as standard; Uronic acid was assayed by the carbazole-sulfuric acid method with glucuronic acid as standard; Glucosamine content was determined by Elson-Morgan method with glucosamine sulfate as standard; Sulfate groups was determined by barium sulfate turbidimetric using potassium sulfate as standard, as previously described (Zhao et al., 2020).

### 2.5 Cellulose acetate electrophoresis

The sample and standard are dissolved by distilled water to prepare 3 mg/mL. The acetate film (8 × 12 cm) was immersed in electrophoresis buffer for 30 min, and the surplus buffer was lightly absorbed by filter paper. Samples were added to the matte surface of the acetate film and electrophoresed at 7 mA for 30 min. Then, the film was dyed in 0.5% Alcian blue-acetic acid for 25 min, and then decolorized with 2% acetic acid for 25 min (Domanig et al., 2009; Wegrowski and Maquart, 2001).

### 2.6 Structural characteristics analysis

#### 2.6.1 FTIR spectroscopic analysis

Samples were dried and then mixed with KBr followed by compression. Then, Fourier transforms infrared (FT-IR) spectra were conducted within the 4000–500 cm^−1^ wavenumber range using a Bruker Tensor 27 FTIR spectrometer.

#### 2.6.2 Molecular weight analysis

The purity and molecular weight (Mw) of the samples were determined by high-performance gel permeation chromatography (HPGPC) on a Waters Ultra hydrogel column 500 (7.8 mm × 300 mm, Japan) and a refractive index detector (Agilent 1200). The Mw of samples was calculated from a standard curve based on reference heparin (Mw: 3500, 5000, 8000, 15000, and 30000 Da) (Yang et al., 2018).

#### 2.6.3 Monosaccharide composition analysis

The M2 monosaccharide composition was detected by 1-phenyl-3-methyl-5-pyrazolone and high performance liquid chromatography (PMP-HPLC). M2 was hydrolyzed with trifluoroacetic acid (TFA, 2 mL) at 110 °C for 6 h and then derivatization was conducted PMP. After reaction ending, the excess PMP was extracted with 2 mL chloroform, and the supernatant was filtered. Then sample was analyzed by Agilen1200 liquid chromatography according to the retention time of monosaccharide standards.

#### 2.6.4 Disaccharide composition analysis

Disaccharide composition of M2 was assessed by strong anion exchange-high-performance liquid chromatography (SAX-HPLC) on waters spherisorb SAX column (4.0 mm × 250 mm, 5 μm, Japan) at 234 nm wavelength, after the sample was enzymatically digested with heparin lyase I, II, III (Pan et al., 2018). Besides, the following disaccharide was used as references: IS, IA, IIS, IIA, IIIS, IIIA, IVS and IVA.

#### 2.6.5 NMR spectroscopy analysis

The M2 sample (50 mg) was soluble in 1 mL of Deuterium oxide (99.9%, Sigma-Aldrich) and performed the ^1^H-NMR,^13^C-NMR, ^1^H-^1^H COSY, ^1^H-^1^H TOCSY, and ^1^H-^1^H NOESY spectroscopy analyses by Bruker Ascend 700M spectrometer at 25 °C.

### 2.7 Mouse bleeding procedure

M2 hemorrhagic effect was induced by transection of the tail in rats (Li, Y, 2006). Rats were randomly allocated into seven groups (5 rats/group). The following groups were considered: (a) rats receiving a tail venous dose of normal saline (10 mL/kg), which served as a control group, (b) rats injected with 10 mL/kg heparin (20 mg/kg) were set as HP group, (c) rats injected with 10 mL/kg sample solution at 5 mg/kg, 10 mg/kg, 20 mg/kg, 40 mg/kg, and 80 mg/kg, were set as the sample group. Thirty minutes after treatment, the mouse tail was cut off at 3 mm from the tip, and the bleeding time of the experimental groups was recorded.

### 2.8 Anticoagulant activity

The samples were formulated with normal saline to prepare solutions of different concentrations (10 μg/mL, 20 μg/mL, 40 μg/mL, 80 μg/mL, and 500 μg/mL). Measurements of PT, APTT and TT were performed according to the manufacturer's recommended protocols (Lindahl et al., 2005).

### 2.9 Fibrinolytic activity

#### 2.9.1 Fibrinolysis *in vitro*

Fibrinolysis *in vitro* was tested using the fibrin plate method (Liu, H et al., 2020; Tage Astrup, 1952). Briefly, 0.3 g agarose powder was dissolved in 20 mL PBS (1M) and autoclaved for 30 min. After the temperature drops to about 55 °C, add thrombin solution (10 U/mL, 2 mL) and fibrinogen solution (0.15%, 10 mL) and mix well, and pour the mixture into a 9 cm culture dish. After it solidifies, some small holes with a diameter of 3 mm were punched and excess water was absorbed. Subsequently, urokinase solution (200 U/mL, 20 μL), PBS solution (1M, 20 μL) as well as samples (1, 6 and 12 mg/mL, 20 μL) were added into the pore respectively. The prepared plates were placed in an incubator at temperature 37 °C for 18 h. The agar plates were then stained with coomassie dye (0.25%) for 30 min and then decolorized with methanol-45% acetic acid solution (45:55). Then, the Dissolving zones’ diameter was determined using a caliper. The calibration curves were made with different concentrations of the urokinases standard (5, 10, 20, 40, 80, and 160 U/mL) and its corresponding ring areas. Finally, fibrinolysis *in vitro* of the sample (12 mg/mL) was computed according to the standard curve and the corresponding dissolving area of the sample.

#### 2.9.2 Fibrinolysis *in vivo*

SD rats were acclimated to the laboratory for at least 7 days prior to surgery or experimentation. They were optionally split into seven groups of 6 rats each and fasted overnight prior to the experimentation. They were anaesthetized with chloral hydrate and injected with samples (1, 6, 12 mg/kg), heparin (1, 6, 12 mg/kg) and saline via the femoral vein. After 2 h of administration, blood was collected by eyeball blood extraction method, coagulated naturally at room temperature for 10–20 min, centrifuged for about 20 min (2000–3000 r), and the upper serum was taken for standby. The levels of serum t-PA, u-PA and PAI-1 in the serum were measured using commercial ELISA kits according to the instructions of the manufacturer (lengton, Shanghai, China)(Duan et al., 2018).

### 2.10 Statistics

The data were expressed as means ± standard deviation (SD) and analyzed using JMP 10 software. A completely randomized design (CRD) was followed for the designing of the experiments and the data were analyzed by ANOVA (analysis of variance).

## 3 Results and discussion

### 3.1 Isolation and chemical properties of heparinoid of M. lusoria

**Fig. 2:**
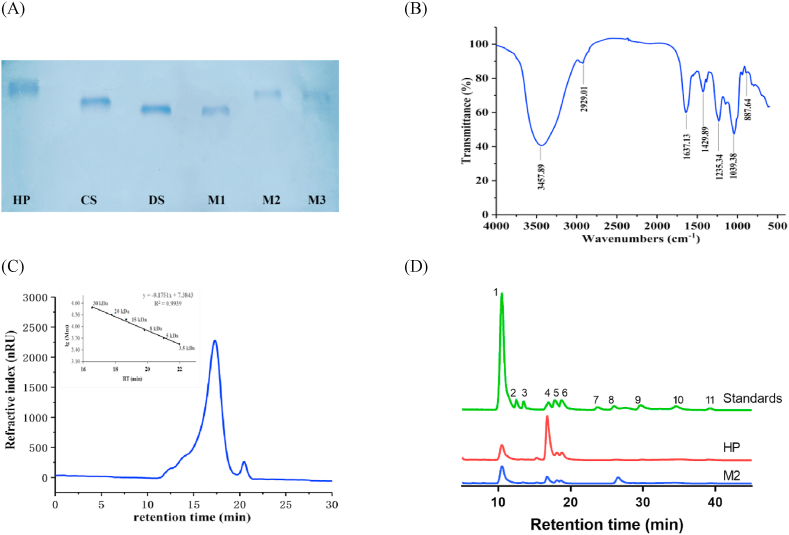
Cellulose acetate electrophoresis of M2 (A); the molecular weight distribution (B); FT-IR spectra(C); Monosaccharide composition of heparinoid M2. The numbered peaks correspond to known monosaccharide standards: 1, PMP; 2, mannose (Man); 3, rhamnose (Rha); 4, glucosamine (GlcN); 5, glucuronic acid (GlcA); 6, iduronic acid (IdoA); 7, N-acetylglucosamine (GlcNAc); 8, glucose (Glc); 9, galactose (Gal); 10, arabinose (Ara), and 11, fucose (Fuc).

Three fractions (M1, M2, M3) were extracted from *M. lusoria* by enzymatic hydrolysis, anion exchange chromatography and alcohol precipitation. Although be obtained from the same clam, there were significant differences in the yield and chemical characteristics of the three fractions (Table 1). The yield of M1, M2 and M3 from *M. lusoria* were about 1.24 mg/g, 0.53 mg/g and 0.18 mg/g, respectively (dry weight). The potency of M2 was about 122.4 U/mg, which was significantly higher than that of M1 (48.0 U/mg) and M3 (85.2 U/mg). The anticoagulant activity of heparin-like compound (M2) from *M. lusoria* was found to be 122.4 U/mg, which was lower than the purified heparin from pig mucosa (180 U/mg) (Valcarcel et al., 2017) and higher than the shrimp heparin (95 U/mg) and crab heparin (33 U/mg). The heparin and uronic acid content of M2 were 614 g/kg and 184 g/kg, respectively, which was higher than that of M1 (506 g/kg and 112 g/kg) and M3 (520 g/kg and 197 g/kg). However, the glucosamine and sulfate groups content of M3 (254 g/kg and 238 g/kg) was higher, when compared with M1 (144 g/kg and 131 g/kg) and M2 (207 g/kg and 148 g/kg). Besides, the protein content of M3 was 21 g/kg, which was lower than that of M1 (128 g/kg) and M2 (58 g/kg), indicating that the purity of M1 was insufficient. In addition, M1 and M2 showed similar electrophoretic mobility to DS and HP, respectively. M3 showed two single bands and similar electrophoretic mobility to HP and CS. (Fig. 2A). Therefore, considering the insufficient purity of M1 and the low extraction rate of M3, M2 was selected for further studies concerning structural characterization and activity.

**Table 1:** Chemical characteristics of heparinoid from *M. lusoria*.

	Anticoagulant potency (U/mg)	Heparin (%)	Uronic acid (%)	Glucosamine (%)	Sulfate groups (%)	Protein (%)
M1	48.0 ± 0.6^c^	50.6 ± 0.1^c^	11.2 ± 1.2^b^	14.4 ± 1.3^c^	13.1 ± 0.1^b^	12.8 ± 0.2^a^
M2	122.4 ± 1.4^a^	61.4 ± 0.3^a^	18.4 ± 0.7^a^	20.7 ± 0.9^b^	14.8 ± 0.3^b^	5.8 ± 0.1^b^
M3	85.2 ± 1.4^b^	52.0 ± 0.2^b^	19.7 ± 0.6^a^	25.4 ± 1.4^a^	23.8 ± 1.0^a^	2.1 ± 0.1^c^

The above percentages are compared with the corresponding dray fraction. Values are means ± SD of 3 parallel measurements. Means with the same letter are not significantly different (*P* > 0.05).

Means with the same letter are not significantly different (*P* > 0.05).

### 3.2 FTIR spectroscopy analysis of M2

The functional groups of M2 were determined by Fourier to transform infrared spectroscopy (Fig. 2B), where the strong absorption peaks appeared at 3457.89 cm^−1^ (O-H band) and 2929.01 cm^−1^ (C-H band) (Li, N et al., 2017). Besides, the band at 1637.13 cm^−1^ was related to the angular oscillation of N-H, and the band at 1429.89 cm^−1^ was the result of the extension oscillation of C-O. The extension oscillation of S=O was represented by a signal at 1235.34 cm^−1^. Furthermore, the signal at 887.64 cm^−1^ indicated that M2 contains β-glycosidic bond, while the signal of 1039.38 cm^−1^ was due to O-H of the pyranose ring (Zhang, 1999).

### 3.3 Molecular weight analysis of M2

The Mw of M2 was determined by HPGPC (Wang, X et al., 2017). In Fig. 2C, M2 shows a wide symmetrical peak and a negligible peak. The retention time of M2 was 17.31 min, corresponding to the Mw of 22.58 kDa. According to previous reports, the Mw of shrimp heparin and crab heparin were 36 kDa and 19 kDa (Chavante et al., 2014; Medeiros, 2000). Besides, the Mw of porcine heparin and bovine heparin were 16 kDa and 25 kDa, respectively. It can be seen that the Mw of heparin/heparinoid varies according to the source.

### 3.4 Monosaccharide composition analysis of M2

The monosaccharide composition of M2 was detected by PMP-HPLC method (Fig. 2D). Glucosamine and glucose were the main components of M2, accounting for 36.57% and 32.40%, and followed by iduronic acid and glucuronic acid, which were 11.59% and 9.18%, respectively. In addition, M2 contained trace amounts of galactose (0.05%), and fucose (0.15%). These results indicated the monosaccharide composition of M2 was closely resemble heparin standard derived from porcine.

### 3.5 Unsaturated disaccharides composition analysis of M2

**Fig. 3:**
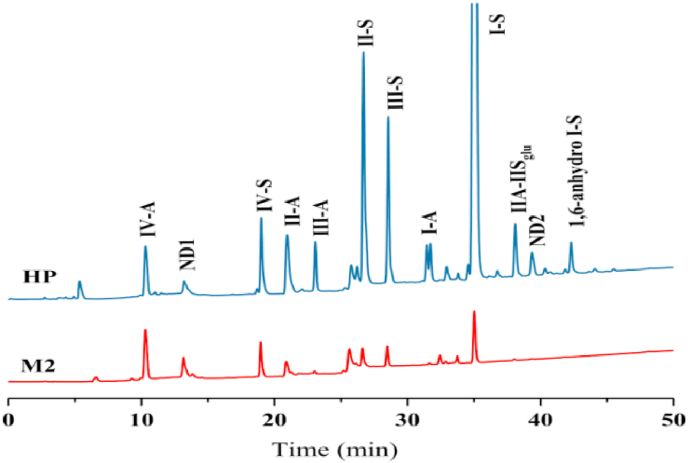
SAX-HPLC chromatograms of unsaturated disaccharide composition in the backbone of M2 and heparin standard (HP). The disaccharide code corresponds to known disaccharide standards: I-S, ΔUA2S-GlcNS,6S; I-A, ΔUA2S-GlcNAC,6S; II-S, ΔUA-GlcNS,6S; II-A, ΔUA-GlcNAC,6S; III-S, ΔUA2S-GlcNS; III-A, ΔUA2S-GlcNAC; IV-S, ΔUA-GlcNS; IV-A, ΔUA-GlcNAC, ND means unknown.

M2 unsaturated disaccharides composition was assessed by strong anion exchange-high-performance liquid chromatography (SAX-HPLC) chromatography (Fig. 3) (Pan et al., 2018). The clam compound were degraded by heparinase (I, II, III), and the products similar to those obtained from the mammalian heparin, although differed in proportions (Table 2). The result showed that M2 was mainly composed of ΔUA2S-GlcNS, 6S (31%), ΔUA-GlcNAC (23%), ΔUA-GlcNS (14%), and a small number of ΔUA2S-GlcNS (9%), ΔUA-GlcNAC, 6S (7%), ΔUA-GlcNS, 6S (8%). The ratios of trisulfated disaccharide, disulfate disaccharide, monosulfated disaccharides and non-sulfated disaccharide in M2 are 34.2: 18.2: 22.0: 25.5, while that of mammalian heparin are 74.7: 18.2: 6.9: 0.2. Thus, an important difference between mammalian heparin and the clam heparinoid is that the level of trisulfated disaccharide in M2 was lower. According to previous reports, prawn heparin and crab heparin also showed this characteristic (Brito et al., 2008; Medeiros, 2000). Thus, we speculated that marine heparin may contains lower amounts of trisulfated disaccharide than that of mammalian heparin.

**Table 2:** Unsaturated disaccharides composition of samples.

Percentage (%)								
	ΔUA2S-GlcNS,6S	ΔUA2S-GlcNAC,6S	ΔUA-GlcNS,6S	ΔUA-GlcNAC,6S	ΔUA2S-GlcNS	ΔUA2S-GlcNAC	ΔUA-GlcNS	ΔUA-GlcNAC
HP	68.18%	1.28%	9.41%	2.71%	6.03%	1.44%	2.13%	<1.00%
M2	31.19%	–	7.63%	7.39%	8.95%	<1.00%	13.87%	23.20%

### 3.6 NMR spectroscopy analysis of M2

**Fig. 4:**
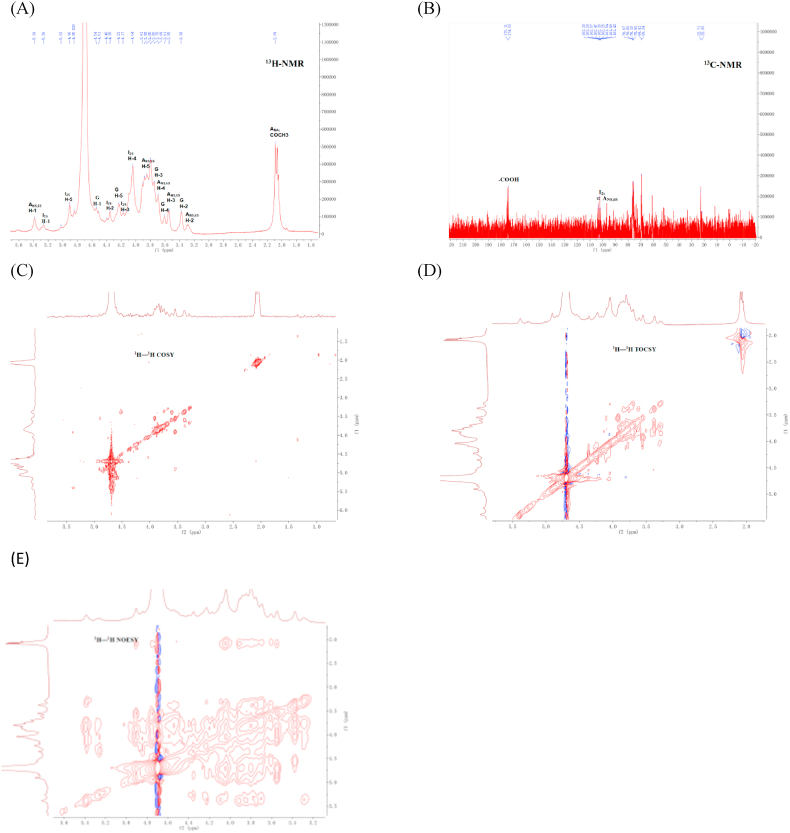
^1^H NMR (A), ^13^CNMR (B), ^1^H-^1^H COSY (C), ^1^H-^1^H TOCSY (D), and ^1^H-^1^H NOESY € of M2, Peaks are assigned as ANS, 6S = GlcNS6S, I_2_S=IdoA2S, G = GlcA, and ANAc = GlcNAc.

As shown in Fig. 4, the molecular framework of M2 was further analyzed by NMR. The ^1^H-NMR spectral signal of M2 is mainly distributed in three regions. The first is the anomeric carbon region ranged from 4.5 to 5.6 ppm, which contains the signal peaks of N, 6-disulfate glucosamine (GlcNS6S, 5.38 ppm), 2-disulfate alduronic acid (IdoA2S, 5.25 ppm), glucuronic acid (GlcA, 4.54 ppm) and IdoA2S (H-5, 4.85 ppm). Besides, there is a peak with the strongest proton signal at 4.70 ppm, which is the solvent peak produced by deuterated water (Du, Z. et al., 2019). The second region is ranged from 2.0 to 2.1 ppm, which is the signal of acetamide methyl. The signal of this region is very strong in the Fig. 4A, indicating the high degree of acetylation of M2. The third region is the proton signal of the sugar ring (3–4.5 ppm).

As shown in Table 3, the signals of each sugar residue were assigned to the signals by referring to the previous literature and combining with NMR spectroscopy (Fu et al., 2013; Monakhova et al., 2018; Palhares et al., 2019). In ^13^C-NMR spectra, the low field signals of 174.04 ppm and 175.03 ppm indicate the presence of carboxyl groups of acetamido and hexuronic acid, and the signal at 22.56 ppm is acetamide methyl carbon. In the ^13^C-NMR spectrum, 90–112 ppm is heterocephalic carbon region. Since the ^13^C-NMR spectrum is obtained by decoupling and each spectral line represents a chemical equivalent carbon atom, so the type of sugar residue can be generally determined by the number of signal peaks in heterocephalic region. There are three anomeric carbon signals in this region at 101.89 ppm, 102.57 ppm and 96.85 ppm, corresponding to the sugar residues IdoA2S, GlcA and GlcNS6S (Yates et al., 1996). Generally, the signals of furanose C3-C5 are distributed at 82–84 ppm, while the signals of pyranose C3- C5 are less than 80 ppm. As shown in Table 3, the C3-C5 chemical signals of IdoA, GlcA and GlcNS6S are all less than 80 ppm, indicating that all three sugar residues are pyranose. In addition, when there is no replacement, the signal distribution of C2 to C4 is between 70–75 ppm, and that of C6 is between 60–64 ppm. When a replacement occurs, the signals of C2-C4 and C6 are moved to 75–85 ppm and 65–70 ppm, respectively (Linqiang Wang, 2013). Therefore, it can be found that C-2 of iduronic acid and C-6 of glucosamine sulfate are substituted (Table 3).

**Table 3:** ^1^H and ^13^C chemical shifts of heparinoid from the clam *M. lusoria*.

Unit	^1^H chemical shifts					
	^1^H/^13^C	^2^H/^13^C	^3^H/^13^C	^4^H/^13^C	^5^H/^13^C	^6^H/^13^C
α-IdoA-2S	5.25/101.89	4.35/79.77	4.17/76.10	4.04/75.88	4.85/69.93	
β-GlcA	4.54/102.57	3.38/73.74	3.84/76.41	3.88/76.65	3.75/80.46	
α-GlcNS-6S	5.38/96.85	3.29/60.78	3.55/73.51	3.69/69.70	3.91/69.17	4.09/67.49

In summary, the sugar residues of M2 were determined to be α-IdoA-2S, β-GlcA, and α-GlcNS-6S by NMR. Among the two kinds of uronic acids, only the iduronic acid had the substitution of sulfuric acid group. Combining infrared spectroscopy, disaccharide composition and one-dimensional nuclear magnetic resonance, the main disaccharide repeat fragments of M2 are: →4)-α-IdoA2S-(1 →4) -α-GlcNS6S-(1→ (31.19%), →4)-β-GlcA-(1→4)-α-GlcNAc(1→ (23.21%), →4)-β-GlcA-(1 →4)-α-GlcNS(1→ (13.87%), →4)-α-IdoA2S-(1 →4)-α-GlcNS(1→ (8.95%), →4)-β-GlcA-(1 →4)-α-GlcNAc6S (1→(7.39%) and →4)-β-GlcA -(1→4)-α-GlcNS6S(1→ (7.63%).

### 3.7 Hemorrhagic activities of M2

**Fig. 5:**
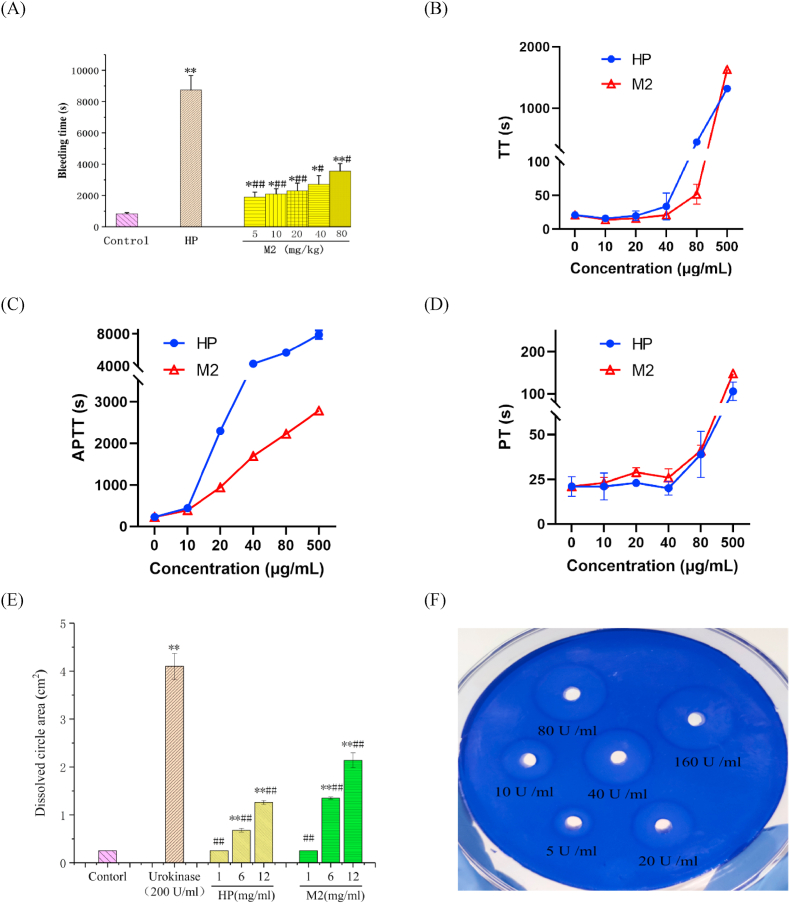
Hemorrhagic effect of M2 (A); Thrombin time (TT) (B); Activated partial thromboplastin time (APTT) (C); prothrombin time (PT) (D); the area of dissolving ring produced by M2 (E); Dissolved circles are produced by different concentrations of urokinase (F). Compared with the control group, *P < 0.05, **P < 0.01; Compared with the Urokinnase group, ^#^*P* < 0.05, ^##^*P* < 0.01.

The clinical application of mammalian heparin was limited by its strong hemorrhagic effect. Therefore, it is necessary to evaluate the bleeding level of heparinoid from *M. lusoria*. As shown in Fig. 5A, HP (20 mg/kg) and M2 groups caused significant hemorrhagic effects compared with the control group (*p* < 0.05). The degree of bleeding in the M2 group was concentration-dependent and lower than that of the HP group (*p* < 0.05). Furthermore, the highest dose group (80 mg/kg) of M2 still showed a hemorrhagic effect about two times lower than that of the HP group. Therefore, heparinoid from *M. lusoria* exhibits a weaker bleeding effect when compared to mammalian heparin. Heparin isolated from an ascidian showed comparable antithrombotic activity in an arterial animal model and lower bleeding effects compared to mammalian heparin (Santos et al., 2007).

### 3.8 Anticoagulant activity of M2

The anticoagulant activity of heparinoid from *M. lusoria* was evaluated by measuring APTT, PT and TT. The three indicators correspond to the intrinsic, extrinsic, and common coagulation pathway, respectively (Gomes et al., 2015). In Fig. 5B. HP could prolong TT at a concentration higher than 40 μg/mL, and M2 prolonged TT at a concentration higher than 80 μg/mL. Fig. 5C showed that APTT was prolonged by HP and M2 in a dose-dependent relationship. Compared with HP, M2 displayed a weaker prolongation effect on APTT. Besides, M2 and HP had no significant difference in the prolongation of PT, and they only extended PT at a concentration higher than 40 μg/mL (Fig. 5D). It turns out that, both M2 and HP made anticoagulant activity primarily by intrinsic and common coagulation pathway, but the anticoagulant activity of M2 was slightly lower than that of HP, which may be due to the low sulfation degree of M2 (Brito et al., 2014; Dietrich et al., 1999; Lindahl et al., 2005). Similarly, previous literature reported that the anticoagulant activity of heparin in bivalve molluscs is higher than that of crustacean heparin and sometimes lower than that of mammalian heparin (Wang, L C et al., 2019). Although the heparin anticoagulant activity of molluscs is lower than that of mammalian heparin, the corresponding bleeding effect is low.

### 3.9 Fibrinolytic activity

#### 3.9.1 Fibrinolysis *in vitro*

Fibrinolysis *in vitro* of M2 was tested using the fibrin plate method. As shown in Fig. 5E, M2 and HP exerted fibrinolytic activity in a dose-dependent relationship. The area of dissolving zones was found to be larger in the HP group (6 and 12 mg/mL) and M2 group (6 and 12 mg/mL) compared with those in the blank control group. The dissolved area of 12 mg/mL M2 and HP were 2.11 cm^2^ and 1.26 cm^2^, respectively. According to the standard curve (y = 0.0576e^1.1674x^, R^2^ = 0.997), which was drawn by the dissolved circle area of different concentrations of urokinase (Fig. 5F), the fibrinolytic activities of HP and M2 were computed to be 0.51 ± 0.02 U/mg and 1.41 ± 0.10 U/mg, respectively. It can be seen that M2 exhibits a fibrinolytic activity about three times stronger than that of HP in the agarose-fibrin plate assay.

#### 3.9.2 Fibrinolysis *in vivo*

Fibrinolysis *in vivo* of M2 was shown in Table 4, u-PA and t-PA could promote the conversion of plasminogen to plasminogen, while PAI could specifically be combined with t-PA in a 1:1 ratio, leading to the deactivation of t-PA. So, t-PA/PAI-1 was possible to indicate the overall situation of the fibrinolytic system (Kohler and Grant, 2000). Similar to the HP group, M2 effectively promoted the secretion of u-PA and t-PA with a dose-dependent relationship (Table 4). For PAI-1, M2 did not show an inhibitory effect on PAI-1, but increased the PAI-1 content, although the effect decreased by concentration. As for t-PA/PAI-1, M2 and HP increase the ratio of t-PA to PAI-1 at 6 mg/mL and 12 mg/mL, indicating theiran active effect on the fibrinolysis pathway. In summary, there were no significant differences of the *in vitr*o fibrinolytic activity between M2 and HP, and they all acted mainly by increasing t-PA and u-PA. *M. lusoria* heparinoid exhibited potential in treatment of thrombotic disease, even though marine heparin/heparinoid seldom used in clinical application (Pavão and Paulo, 2012).

**Table 4:** Effects of M2 on Rats fibrinolytic system.

Index	Injection	Concentration (mg/kg)			
		0	1	6	12
t-PA (ng/mL)	HP	23.86 ± 3.80 ^d^	46.97 ± 5.07 ^a^	65.24 ± 10.75 ^a^	65.72 ± 4.23 ^a^
	M2	23.86 ± 3.80 ^d^	36.31 ± 2.98 ^c^	37.48 ± 6.18 ^b c^	43.45 ± 2.12 ^b c^

u-PA (ng/mL)	HP	1.12 ± 0.06 ^b c^	1.01 ± 0.07 ^c^	1.23 ± 0.09 ^a b c^	1.41 ± 0.14 ^a^
	M2	1.12 ± 0.64 ^b c^	1.01 ± 0.13 ^c^	1.34 ± 0.23 ^c^	1.51 ± 0.03 ^a^

PAI-1 (ng/mL)	HP	12.80 ± 1.57 ^c^	37.50 ± 17.56 ^a^	33.21 ± 11.95 ^a b^	24.05 ± 5.93^b c^
	M2	12.80 ± 1.57 ^c^	24.34 ± 5.11 ^a b c^	19.74 ± 1.56 ^b c^	19.39 ± 0.47 ^b c^

t-PA/PAI-1	HP	1.86 ± 0.07	1.25 ± 0.68	1.96 ± 0.39	2.73 ± 0.31
	M2	1.86 ± 0.07	1.50 ± 0.19	1.90 ± 0.16	2.54 ± 0.06

Values represent mean ± SD, Means with the same letter are not significantly different (*P* > 0.05).

In general, thrombosis is a complex physiological process that is caused by many factors, including clotting of the blood in the vessel system and fibrin protein clot that usually leads to severe health issues. Due to mammalian heparin expose some clinical risks, it is crucial to locate reliable and accessible sources to replace mammalian heparin, marine-derived heparin might be a good choice. In this study, a natural heparinoid (M2) from *Meretrix lusoria* was found to play an antithrombotic activity through anticoagulant and fibrinolytic effects. On the one hand, M2 inhibited clotting through intrinsic and common pathways, which was similar to porcine heparin. Thus, we speculated that the anticoagulant mechanism of M2 is resemble with heparin, which may relate to antithrombin and heparin cofactorⅡ. Similar results were reported that molluscs heparin exhibits the better anticoagulant effect than mammalian heparin, and its mechanism is also related to binding antithrombin Ⅲ(Valcarcel et al., 2017). For another thing, M2 exhibited pretty antithrombotic activity comparable to porcine heparin and lower bleeding side effect *in vivo*. Their biological activities appear influenced by the different structure interactions of M2 and mammalian heparin.

Heparin in marine species has been found to differ from heparin in mammalian organisms, primarily in terms of molecular weight and sulfation pattern, and to have distinct structures (Kozlowski et al., 2011). The disaccharide makeup of the heparins from differ species is generally quite diverse, with varying proportions of the following units: IdoA(2S)-GlcNS(6S), IdoA-GlcNS(6S), IdoA(2S)-GlcNS, IdoA-GlcNS, GlcA-GlcNS, GlcA-GlcNAc. The present study demonstrate these findings, and M2 also different from mammalian heparin. An important difference is that the level of trisulfated disaccharide in M2 was lower, the ratio of trisulfated disaccharide in M2 is 34.2, while that of mammalian heparin is 74.7. In terms of the activity difference, mammalian heparin standards exhibit greater APTT activity at the same dose than M2. Another difference in activity was that the hemorrhagic tendency was significantly lower in M2 than in mammalian heparin. This is influenced by the sulfation pattern, may because of the presence of specific disaccharide sequences. It is still further research on M2 regarding its detail structure, antithrombotic mechanisms, and other effects.

## 4 Conclusions

It was concluded that M2 is a polysaccharide mixture with similar characteristics to heparin. The extraction rate of M2 is 0.53 mg/g (dry weight) and the average molecular weight is 22.58 kDa. Besides, it is majorly composed of →4)-α-IdoA2S-(1 →4)-α-GlcNS6S-(1→ (31.19%), →4)-β-GlcA-(1 →4)-α-GlcNAc (1→ (23.21%), →4)-β-GlcA-(1 →4)-α-GlcNS (1→ (13.87%), →4)-α-IdoA2S-(1 →4)-α-GlcNS (1→ (8.95%), →4)-β-GlcA-(1 →4)-α-GlcNAc6S (1→(7.39%) and →4)-β-GlcA-(1 →4)-α-GlcNS6S(1→ (7.63%). The anticoagulant potency of *M. lusoria* heparin analogue is 122.4 U/mg, which plays an anticoagulant role primarily mediated by intrinsic and common coagulation pathway. In addition, the fibrinolytic activity of *M. lusoria* heparin analogue is 1.41 U/mg, which mainly through promoting the release of t-PA. Although the anticoagulant activity of heparinoid from *M. lusoria* is lower compared with that of mammalian heparins, the hemorrhagic reaction caused by the analogue is much lower than that of mammalian heparins. The fibrinolytic activity of *M. lusoria* analogue is slightly higher than that of mammalian heparins. Therefore, heparinoid from *M. lusoria* exposes mild anticoagulant activity, low bleeding effect and strong fibrinolytic activity. M2 can be considered as an alternative for using mammalian heparin, and developing functional foods or drugs with antithrombotic effects.

## CRediT authorship contribution statement

**Jing Chen:** Conceptualization, Methodology, Data curation, Formal analysis, Writing – original draft. **Zhenxing Du:** Conceptualization, Methodology, Data curation, Formal analysis, Writing – original draft. **Bingbing Song:** Methodology, Writing – review & editing. **Rui Li:** Methodology, Writing – review & editing. **Xuejing Jia:** Visualization. **Jianping Chen:** Methodology, Writing – original draft. **Xiaofei Liu:** Methodology, Writing – review & editing. **Saiyi Zhong:** Methodology, Software, Writing – review & editing, Supervision, Project administration, Funding acquisition.

## Declaration of competing interest

The authors declare that they have no known competing financial interests or personal relationships that could have appeared to influence the work reported in this paper.
